# Shaping the Future of HTA in Italy: Insights from the Italian Health Policy Forum

**DOI:** 10.3390/jmahp13040047

**Published:** 2025-09-24

**Authors:** Paolo Sciattella, Roberta Laurita, Chiara Bini, Eugenio Di Brino, Dario Sacchini, Giandomenico Nollo

**Affiliations:** 1 Centre for Economics and International Studies-Economic Evaluation and Health Technology Assessment, Faculty of Economics, University of Rome “Tor Vergata”, Via Columbia 2, 00133 Rome, Italy; paolo.sciattella@uniroma2.it (P.S.); chiara.bini@uniroma2.it (C.B.); 2 Health Policy Forum, SIHTA (Italian Society of Health Technology Assessment), 00144 Rome, Italy; eugenio.dibrino@unicatt.it; 3 Department of Management, Faculty of Economics, Università Cattolica del Sacro Cuore, Via Necchi, 7, 20123 Milan, Italy; 4 ALTEMS, Università Cattolica del Sacro Cuore, Largo Francesco Vito 1, 00168 Rome, Italy; 5 Research Center for Clinical Bioethics and Medical Humanities, Department of Healthcare Surveillance and Bioethics, Università Cattolica del Sacro Cuore, Largo Francesco Vito 1, 00168 Rome, Italy; dario.sacchini@unicatt.it; 6 Italian Society of Health Technology Assessment (SIHTA), Via Massimo Bontempelli 10, 00144 Rome, Italy; giandomenico.nollo@unitn.it; 7 Department of Industrial Engineering, University of Trento, Via Sommarive, 9, 38123 Trento, Italy

**Keywords:** health technology assessment, EU-HTA Regulation, medical devices, medicinal products

## Abstract

The implementation of the European (EU) Health Technology Assessment (HTA) Regulation 2021/2282 (EU HTAR) offers many opportunities, aimed at harmonizing HTA procedures and improving access to innovations; it also represents a significant challenge for the European healthcare system. Within the 2024 Health Policy Forum Italy meeting, different actors, stakeholders, and institutions had the opportunity to discuss major criticism and opportunities coming from the EU-HTA Regulation addressing future developments in the healthcare sector. Two groups, EU & Italy Pharmaceuticals and EU-Italy Medical Devices, worked distinctively on the EU HTAR by highlighting key issues that may pose challenges at both European and national levels, proposing potential solutions. The allocation of participants into two groups, according to their affiliation with either the pharmaceutical or the medical device sector, enhances the diversity of professional backgrounds and institutional perspectives, thereby fostering a more comprehensive and informed discussion. The recommendations highlighted by the two groups emphasize the need to promote cooperation among Member States, strengthen training for decision-makers, and develop a monitoring system to evaluate EU HTA’s impact.

## 1. Introduction

Health technologies that are new to the market undergo a process, called health technology assessment (HTA), aimed at evaluating direct and indirect consequences through a systematic and multidisciplinary evaluation. This instrument provides a summary of major information concerning clinical, economic, organizational, social, legal and ethical domains related to the use of health technologies. To make the process efficient, all this information needs to be considered in the specific context in which the technology is going to be used. Thus, discrepancies arising from differences coming from differences in national healthcare systems and willingness to invest in healthcare is difficult to manage [1]. The direct effect is the creation of disparities in terms of patients’ access to treatments. In December 2021, the Health Technology Assessment Regulation (EU) 2021/2282 (EU HTAR) has been adopted establishing common standards for evaluating health technologies (medicinal products, Class IIb and III medical devices and class D in vitro diagnostics) in the European Union, aiming to harmonize processes and ensure equitable access to innovations [2,3]. Furthermore, by harmonizing methodological standards for HTA it also fosters collaboration among the European HTA bodies.

Nevertheless, the effectiveness of the EU HTAR depends on addressing key challenges that call for cooperation, flexibility, and investment in training, in order to ensure access to quality care while fostering innovation across the EU. In doing so, it is necessary to ensure coordination among different actors: Member States, European institutions, HTA bodies, and developers and other healthcare stakeholders [4,5]. In fact, fostering dialog, flexibility, and cooperation is essential to provide harmonization, effectiveness, and fairness for the European HTA Regulation.

A central principle of the Regulation is that JCAs will focus exclusively on clinical domains—relative effectiveness and safety—while leaving to Member States the appraisal of non-clinical aspects, including cost-effectiveness, budget impact, ethical, organizational, and social considerations [3]. This division of labor is designed to safeguard national sovereignty over pricing and reimbursement decisions, while simultaneously providing a robust, shared scientific foundation for these deliberations. Consequently, national authorities are expected to “give due consideration” to JCA reports when conducting their own HTA, a provision that implies integration without full substitution.

The EU HTAR came into effect in January 2022, marking the start of a three-year transition period, the so called “preparation phase”. Starting from January 2025, joint clinical assessments (JCAs) will be conducted for all cancer medicines and advanced therapy medicinal products (ATMPs), followed by medical devices from 2027, orphan drugs from January 2028, and finally all other centrally approved medicines included vaccines from 2030 onwards (Article 7.2) [3]. In this period of transition, different key activities have been undertaken to successfully prepare the implementation of the Regulation: the formation of the Coordination Group among member states, with its subgroups on JCAs, Joint Scientific Consultations (JSCs); the Identification of Emerging Health Technologies and Methodology; the establishment of the Stakeholder Network, generating Guidance Documents and drafting of the Implementing and Delegated Acts [2].

For Italy, where HTA capacity has developed unevenly across regions, the Regulation presents both opportunities and challenges. On the one hand, the availability of standardized, high-quality JCA reports can support greater consistency in national and regional evaluations, reduce administrative burden on health technology developers, and accelerate decision-making for market access. On the other hand, the Italian system will need to adapt its appraisal frameworks to effectively incorporate JCA evidence, ensuring that country-specific economic and organizational factors are adequately captured. This adaptation will require careful methodological alignment, investments in training of evaluators, and possibly the revision of existing HTA processes at AIFA and at regional level to avoid redundancy.

## 2. Methodology

### Health Policy Forum

The Health Policy Forum (HPF) is an initiative of the Italian Society of Health Technology Assessment (SIHTA), inspired by the Global Policy Forum provided by the International Society of HTA (HTAi), and represents a continuous and structured opportunity for discussion between professionals operating in the field of HTA, belonging to Institutions, Regions, Industry, Patient Associations, Scientific Societies and Universities. The aim is to contribute to the technical and scientific debate on HTA methods and to develop knowledge in the field of HTA in Italy.

The here presented comments are a summary of the discussions held at the 2024 Health Policy Forum-Italy meeting. The meeting was conducted under the Chatham House Rule [6], whereby participants are free to share information without revealing their identity or affiliation.

Within the 2024 HPF, different institutions’ representatives, stakeholders and patient associations had the opportunity to discuss major criticisms and opportunities coming from the EU-HTA Regulation addressing future developments in the healthcare sector, with a focus on clinical domains. The introductory remarks of the HPF emphasized the fundamental role of EU HTAR in promoting informed healthcare decisions, focusing Member States’ attention on three main aspects: harmonization of assessment procedures, equitable access to health technologies, and transparency of HTA reports.

Two groups, EU & IT Pharmaceuticals and EU-Italy Medical Devices, worked distinctively on the EU HTAR by highlighting key issues that may pose challenges at both European and National levels, proposing potential solutions and/or mitigations. By considering both perspectives, the starting point is the fact that the EU HTAR is a crucial process for evaluating health technologies and informing their accessibility in the European healthcare system.

Each group consisted of 20 participants with diverse roles and levels of seniority, ensuring that the discussion benefited from multiple perspectives. The session began with preliminary questions, which were debated based on participants raising their hands, while the chairman carefully took notes. At the end, the chairman presented a final slide summarizing all the inputs and facilitating consensus among all participants. Final comments and feedback are provided in the plenary session where all the stakeholders and representatives of the main institutions met together.

## 3. Results

The recommendations from each group should be interpreted within the context of the participants’ backgrounds. Since the groups were composed considering their specific expertise, the discussions and resulting recommendations are valid primarily within the context of that group and cannot be readily generalized to the other domain. Therefore, the first group’s recommendations pertain to pharmaceuticals, while the second group’s recommendations pertain to medical devices and IVDs. Furthermore, in Italy this is necessary because of the presence of two institutions (AIFA and AGENAS) in charge of medicinal products and medical devices that work together under the Ministry of Health.

The results indicate that, unlike regulatory assessments focused on the overall benefit–risk ratio, Health Technology Assessment (HTA) emphasizes relative clinical effectiveness and safety. This distinction between HTA and regulatory processes creates challenges, prompting Health Technology Developers (HTDs) to pursue scientific consultations to generate evidence suitable for both pathways. Strengthening institutional capacity at both European and national levels, together with enhanced collaboration among Member States, is recommended to support evidence generation, share results, and optimize resources.

### 3.1. Policy Recommendations for Medicinal Products for Human Use

The EU & Italian Pharmaceutical group highlighted five main points that need a particular attention:

- The selection of PICOs should be informed by diverse local perspectives, initially drawing upon European-level position papers that integrate the views of a wide range of stakeholders, including, for instance, European scientific societies. Ultimately, however, responsibility should remain with the HTA agencies of the individual Member States, as they alone are positioned to adequately reflect the specific features and priorities of their respective national contexts.
- Strengthening study evaluations based on a shared European hierarchy of evidence (a starting point is the Guidelines issued on the validity of clinical studies) [7].
- Stakeholder training: The regulation establishes a network of stakeholders to support the work of the HTA Coordination Group (HTACG) and encourages information exchanges and annual meetings. It is hoped that universities and scientific societies will continue providing training for HTA decision-makers. Locally, structured pathways are needed for national decision-makers and experts. Expanding the number of HTA experts is essential for quality assessment and consultation on national PICOs.
- Monitoring EU HTAR is vital to ensure efficiency and cost-effectiveness. The European Commission will need to report on progress made in terms of healthcare system sustainability, access to innovative technologies, and cost reduction. A monitoring dashboard to collect data on the impacts of EU HTAR adoption could improve transparency and access to relevant information for Member States and particularly Italy.
- Finally, national access remains a critical issue; although the JCA aims to harmonize assessments, a strong connection with the national and regional process (e.g., in countries such as Italy or Spain) is recommended to ensure accelerated access to therapies.
- Considering the national context, in Italy it is needed to include all the non-clinical domains in the HTA Body Negotiation Procedure assigning a specific value to the organizational impact and ELSI (ethical, legal and social issue) domains.

### 3.2. Policy Recommendations for Medical Devices and In Vitro Diagnostic Medical Devices

The EU-Italy Medical Devices Working Group’s discussion focused on the implementation of the EU HTAR and its impact at the national level in relation to the National HTA Program for Medical Devices by Italian National Agency for Regional Healthcare Services (AGENAS) [8]. Medical devices present unique characteristics: broad heterogeneity, short life cycles with continuous innovations, the need to adapt to the operator’s experience level, and the context of use. These specifics require flexible assessment processes and methodologies tailored to each device type, such as implantable, diagnostic, and digital devices.

Among the group’s proposals:

- Horizon Scanning (HS): This system enables the timely identification of emerging technologies that could significantly impact healthcare. Stakeholders may suggest evaluating technologies with major impacts based on parameters like disease burden and equity of access, opening a discussion with Emerging Technologies Subgroup.
- At national level, increased stakeholder involvement in the HTA process is needed. This should be facilitated through early dialog with different stakeholders and HTDs.
- Adaptability of the JCA to the nature of medical devices which requires a different integration between the JCA and the CE marking requirements is necessary to ensure predictability for manufacturers.
- Timeliness of selection: A timely JCA process is crucial to improve access to new technologies. Coordinating the pre-CE mark process with the JCA is recommended to avoid delays in device access.

## 4. Discussion

The implementation of the HTA Regulation (EU) 2021/2282 represents a significant challenge for the European healthcare system, aimed at harmonizing HTA procedures and improving access to innovations [2,3]. To transform this opportunity into tangible benefits for citizens and healthcare systems, decisive and targeted actions are essential. This process requires coordinated collaboration among Member States, healthcare institutions, developers and SHs. International experience suggests that successful implementation depends on robust governance, clear communication channels, and proactive stakeholder engagement [9].

In Italy, as in the other Member States, the effective implementation of this regulation will be essential to ensure timeliness and quality of healthcare decisions. Furthermore, structured dialog among stakeholders, transparency, and flexibility will be key to avoiding delays in accessing innovative technologies [9]. For Italy, where patient advocacy groups are increasingly recognized as essential contributors to decision-making, the EU HTAR could serve as a catalyst for more structured involvement of patients and civil society in national HTA. In a recent study, an interim evaluation of the preparation phase of the EU HTA Regulation was conducted [5]. Their analysis highlighted critical priorities, including harmonization, capacity building, and uncertainty management, with specific attention to the adoption of living guidelines, the clarification of the PICO process, and the alignment of national policies to enable effective implementation by 2025. Elaborating on these findings, the present analysis emphasizes the necessity of structured training programs for key stakeholders (clinicians, patients, HTA bodies, and payers) and underscores the importance of preventing duplication and inconsistencies in PICO development across Member States. In fact, the recommendations highlighted by the two groups emphasize the need to promote cooperation among Member States, strengthen training for decision-makers, and develop a monitoring system to evaluate EU HTAR’s impact. Additionally, it is crucial that the HTA process adapts to the unique characteristics of medical devices, considering their specific features, such as short innovation cycle. In this regard, it is essential to foster close collaboration with the Coordination Group, capitalizing on the Joint Scientific Consultation, which represents the most effective instrument for aligning evidence generation with the development of the technology. Likewise, ensuring rapid and equitable access to healthcare technologies by reducing assessment timelines will be essential. It is also recommended to integrate non-clinical domains into the national negotiation process so that assessments consider economic, organizational, ethical, legal, and social aspects.

Finally, the Regulation underscores the need for sustained capacity building. The Italian HTA system will face challenges of human resource allocation, methodological expertise, and sustainable financing. Aligning with the EU framework may require Italy to invest in specialized skills such as indirect treatment comparisons, network meta-analysis, and horizon scanning. At the same time, Italy can leverage its participation in European HTA cooperation to access expertise and share best practices, ultimately reinforcing its national capacity. In conclusion, the EU HTAR offers Italy an unprecedented opportunity to modernize its HTA processes, enhance alignment with European standards, and strengthen the scientific basis of its policy decisions. However, realizing these benefits will depend on timely institutional reforms, effective stakeholder coordination, and a proactive strategy to integrate JCA evidence into the broader national appraisal framework. The Italian experience in adapting to the EU HTAR will not only shape patient access to innovation domestically but also contribute to the collective European endeavor of building a coherent, efficient, and equitable system for health technology assessment.

These recommendations are not merely a call to action, but they are a suggestion to harmonize healthcare systems in Europe, making them more innovative, and more resilient. Their implementation is a unique opportunity to demonstrate that Europe is ready to meet future challenges with boldness and strategic vision, aiming at promoting innovation, equity, and sustainability.

## Data Availability

No new data were created or analyzed in this study.
